# Development of the support needs after ICU (SNAC) questionnaire

**DOI:** 10.1111/nicc.12695

**Published:** 2021-08-13

**Authors:** Brenda O'Neill, Mark Linden, Pam Ramsay, Alia Darweish Medniuk, Joanne Outtrim, Judy King, Bronagh Blackwood

**Affiliations:** ^1^ Centre for Health and Rehabilitation Technologies Ulster University Newtownabbey Northern Ireland UK; ^2^ School of Nursing and Midwifery Queen's University Belfast Belfast Northern Ireland UK; ^3^ School of Health Sciences University of Dundee Dundee Scotland UK; ^4^ Department of Anaesthesia Southmead Hospital, North Bristol NHS Trust Bristol UK; ^5^ Division of Anaesthesia, Department of Medicine University of Cambridge Cambridge UK; ^6^ School of Rehabilitation Sciences University of Ottawa Ottawa Ontario Canada; ^7^ Wellcome‐Wolfson Institute for Experimental Medicine Queen's University Belfast Belfast Northern Ireland UK

**Keywords:** adult intensive care, ICU follow‐up, questionnaire design/survey

## Abstract

**Aims:**

To develop a questionnaire to identify Intensive Care survivor needs at key transitions during the recovery process, and assess its validity and reliability in a group of ICU survivors.

**Methods:**

Development of the **S**upport **N**eeds **A**fter I**C**U (SNAC) questionnaire was based on a systematic scoping review, and analysis of patient interviews (n = 22). Face and content validity were assessed by service users (n = 12) and an expert panel of healthcare professionals (n = 6). A pilot survey among 200 ICU survivors assessed recruitment at one of five different stages after ICU discharge [(1) in hospital, (2) < 6 weeks, (3) 7 weeks to 6 months, (4) 7 to 12 months, or (5) 12 to 24 months post‐hospital discharge]; to assess reliability of the SNAC questionnaire; and to conduct exploratory data analysis. Reliability was determined using Cronbach's alpha for internal consistency; intraclass correlation coefficients for test–retest reliability. We explored correlations with sociodemographic variables using Pearson's correlation coefficient; differences between questionnaire scores and patient demographics using one‐way ANOVA.

**Results:**

The SNAC questionnaire consisted of 32 items that assessed five categories of support needs (informational, emotional, instrumental [e.g. practical physical help, provision of equipment or training], appraisal [e.g. clinician feedback on recovery] and spiritual needs). ICU survivors were recruited from Northern Ireland, England and Scotland. From a total of 375 questionnaires distributed, 202 (54%) were returned. The questionnaire had high internal consistency (0.97) and high test–retest reliability (r = 0.8) with subcategories ranging from 0.3 to 0.9.

**Conclusions:**

The SNAC questionnaire appears to be a comprehensive, valid, and reliable questionnaire. Further research will enable more robust examination of its properties e.g. factor analysis, and establish its utility in identifying whether patients' support needs evolve over time.

**Relevance to clinical practice:**

The SNAC questionnaire has the potential to be used to identify ICU survivors' needs and inform post‐hospital support services.

**What is known about the subject**

- A key priority for the intensive care community is to identify how and when to support ICU survivors at key transitions in care.

**What this paper contributes**

- This new **S**upport **N**eeds **A**fter I**C**U (SNAC) questionnaire has the potential to inform the development of support services for ICU survivors.
- Future research using the SNAC questionnaire following the same group of patients across their recovery continuum would identify whether patients' support needs evolve over time.

## 1 BACKGROUND

The need for support throughout the post‐ICU recovery process has long been identified.^1^, ^2^, ^3^, ^4^ A key priority for the intensive care community is determining how and when to support ICU survivors and their families/carers.^5^, ^6^ Guidelines recommend that individualized assessment, rehabilitation, and multidisciplinary follow‐up services should be provided,^7^, ^8^ but there is little consensus on the components, individualization, timing, mode of delivery (e.g. face to face, telephone or online), and duration of such services.^9^, ^10^, ^11^, ^12^, ^13^, ^14^ Furthermore, there are no standardized, validated tools to evaluate the extent to which these services, where they exist, meet patients' needs. We aimed to address this gap, by developing a support needs questionnaire for intensive care survivors which could be used to assess patient identified needs at key transitions during the recovery process.

### 1.1 Support needs

Support needs have been defined as “the additional help some adults need in order that they can live in the best way they can, despite any illness or disability they might have.”^15^ Support needs questionnaires have been developed for other populations and these can help to shape service provision and co‐ordination, and enhance a patient centred approach to care.^16^, ^17^, ^18^, ^19^, ^20^, ^21^ The Social Support Needs framework developed by House categorized needs as informational, emotional, instrumental (e.g. provision of practical physical help, training or equipment), and appraisal (e.g. reassurance, clinician feedback on recovery), which may be short‐ or long‐term and/or evolve across the recovery trajectory.^2^, ^15^, ^22^

We previously conducted a scoping review of the qualitative literature on ICU survivors' support needs^15^ and identified the Timing it Right framework (TIR)^23^, ^24^ as a useful and relevant means of capturing ICU survivors' support needs during the different stages of recovery. The TIR framework was initially developed for use among family members of stroke survivors,^23^, ^24^ and it provides a practical, time‐based framework in which survivors' needs during recovery can be explored. The TIR framework has also been used among ICU survivors^4^ to describe key transition phases across the recovery continuum: from the event in ICU, to stabilization on the hospital ward, preparation for hospital discharge, the early phase at home, and longer‐term recovery (adaptation phase).^4^ This study is framed around the recovery stages that align to the TIR framework (excluding the event in ICU).^4^, ^15^ Recovery after a stay in ICU is highly individualized,^1^, ^15^ and it is difficult to anticipate patients' support needs at key stages across the recovery trajectory. An assessment tool to capture ICU patients' needs at various time points across their recovery trajectory may help to streamline patient care by highlighting the support services needed at particular times.

The main aim of this study was to develop a **S**upport **N**eeds **A**fter I**C**U (SNAC) questionnaire to identify intensive care survivor needs at key transitions during the recovery process, and assess its validity and reliability in a group of ICU survivors.

Objectives were to:

1. Develop and establish face and content validity (interviews, scoping review, service user and expert panel assessment) of SNAC.
2. Conduct a pilot survey to
  - Assess recruitment and response rates from ICU survivors at key stages of recovery after ICU (to inform future use of SNAC for practice and research)
  - Assess the questionnaire's reliability (consistency and test–retest reliability)
  - Conduct exploratory analysis of associations between sociodemographic data and SNAC results to generate potential hypotheses for testing in future work.

## 2 METHODS

**FIGURE 1:**
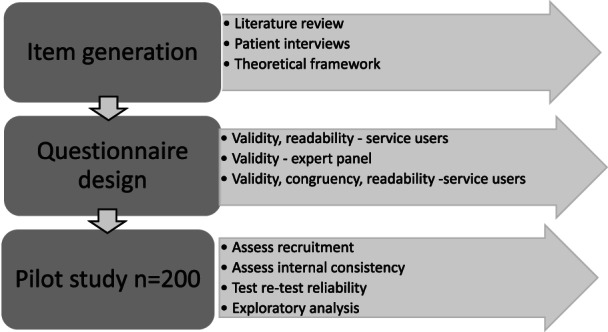
Summary of the SNAC Questionnaire development process

The study was conducted in two stages: 1. questionnaire development and assessment of validity; and 2. a pilot survey to assess recruitment of ICU survivors, reliability of SNAC, and exploratory analysis of data (Figure 1). Ethical approval was granted from the Office for Research Ethics Northern Ireland 17/NI/0236 and research governance for individual collaborating sites was obtained. Completed and returned questionnaires were accepted as informed consent. Questionnaires were coded to ensure confidentiality and all data were stored on password protected computers in accordance with the Ulster University research procedures.

### 2.1 Stage 1. Questionnaire development and assessment of validity

The approach to developing and establishing face and content validity of the SNAC questionnaire followed key recommendations for developing questionnaires.^25^, ^26^, ^27^, ^28^, ^29^

#### 2.1.1 Item generation

Items were generated from three sources: detailed findings from a longitudinal qualitative study of ICU survivors' support needs during the year post‐ICU discharge^30^; a published scoping review of 32 qualitative studies^15^; and iterative consensus meetings among the multidisciplinary research team.

The longitudinal qualitative study was undertaken previously by a co‐author (PR) among adult ICU survivors who were mechanically ventilated for ≥2 days and discharged from one of two Scottish ICUs.^30^ Interviews were undertaken at four time points: prior to hospital discharge, and at 4 to 6 weeks, 6, and 12 months following ICU discharge. These time points align to the TIR recovery phases including stabilisation and preparation for hospital discharge, and the early and longer‐term home recovery. Using thematic analysis, data were coded with reference to patient support needs identified in the TIR framework. Findings showed that support needs commonly included making sense of the critical illness experience (including illness severity, amnesia, dreams, hallucinations, flashbacks); making sense of physical, psychological, and emotional frailty; a perceived lack of understanding among clinicians; social isolation following hospital discharge; vulnerability; concern for family members; and the socioeconomic burden of critical illness.^30^

The scoping review included published qualitative research from 2000 to 2017 reporting on adult ICU survivors' support needs.^15^ Two reviewers (JK, PR) independently screened, extracted and thematically analysed the research themes with reference to the support needs identified in the TIR framework. Informational, emotional, instrumental, appraisal, and also spiritual support needs were identified, and the nature and intensity of need differed when mapped against the time‐points of the TIR framework.^15^

The initial questionnaire (version 1.0, n = 31 items) was developed by the multidisciplinary research team with clinical and research expertise in patient recovery, support, and rehabilitation after ICU (n = 6). The team comprised medicine and anaesthesia (ADM), nursing (BB, PR), physiotherapy (BO'N, JK), and psychology (ML).

#### 2.1.2 Questionnaire design, face and content validity, readability

The design of the questionnaire progressed using an iterative process involving the research team, ICU service users (n = 12) and an expert health care professional group (n = 6) independent to the research team.

Using a predesigned proforma (Supplementary Figures/Tables), the team initially sought feedback on the questionnaire's face and content validity, readability, understanding, and user friendliness from: ICU service users (former patients) (n = 6) recruited from UK ICU support groups and follow up clinics; and from health care professionals specifically recruited for their expertise in the field (n = 6, General Practitioner, ICU Intensivist, Clinical Psychologist x 2, Specialist follow‐up Nurse, Specialist Physiotherapist). Using an iterative process, the research team collated and reviewed the feedback, and amended the questionnaire. Additional ICU service users (n = 6) commented on questionnaire readability and understanding. They also assessed item congruence with the aim of the questionnaire, by rating the importance of each item using a scale (1, very important, this item should be included in the questionnaire; 2, somewhat important, it would not matter if this item was in or out of the questionnaire; 3, not important, this item should be removed)^32^ (Supplementary Figures/Tables). To further assess readability, we used Drivel Defence software^33^ to optimise the number of words in each sentence and enhance comprehension.

#### 2.1.3 Data analysis

Feedback from the ICU service users and health professionals was collated and reviewed by the research team. The team discussed items that needed changed, added, reduced or wording altered and consensus was reached regarding any amendments. This process was repeated following further review by the additional ICU service users. For congruency, the average percentage agreement between service users for each question in the SNAC questionnaire was calculated. The Drivel Defence Index^33^ provides an automated calculation of the number of words in a statement or sentence; any statement with more than 20 words was reviewed and amended.

### 2.2 Stage 2. Pilot survey to assess recruitment of ICU survivors at key stages of recovery, reliability of the SNAC questionnaire, and conduct exploratory analysis of data

#### 2.2.1 Participants and setting

We recruited general ICU survivors from four UK hospitals and from a UK patient support group, ICU Steps (www.ICUsteps.co.uk). We included participants at five time points aligned to the TIR framework: (1) on the hospital ward; (2) discharged from hospital within 4 to 6 weeks; (3) at home between 7 weeks to 6 months; (4) between 7 to 12 months; (5) or 12 to 24 months.

Questionnaires were administered by post, by dedicated research staff in each setting. Inclusion criteria comprised adults over 18 years old with an ICU admission in the previous 12 months requiring mechanical ventilation for ≥48 hours. We excluded survivors requiring palliative care, planned specialist support pathway (e.g. liver transplant), with a neurological event (e.g. head injury or neurodegenerative condition), or if they declined or were unable to give consent. The return of the completed questionnaire was accepted as consent.

#### 2.2.2 Sample size

A formal sample size calculation is not required during pilot testing.^34^ However, we aimed to recruit 200 participants from the four sites to obtain preliminary data for the iterative refinement of the questionnaire, to ensure sufficient data to assess adequacy of our analysis procedures, and to inform ability to recruit ICU survivors. The sample included representation from across the continuum of recovery after ICU i.e. ward, 4 to 6 weeks post discharge, 6 months, and 12 months. We estimated 50 participants of the total sample would be acceptable to complete the questionnaire on a second occasion for the test retest reliability. This was based on recommendations that a minimum of 22 participants would be required to detect a medium sized intraclass correlation coefficient (ICC) of 0.5.^31^ We over‐inflated the number to ensure that any smaller ICCs were detectable.

#### 2.2.3 Recruitment procedure

Patients were identified from ICU registers/databases by dedicated staff at each of the participating sites, or via email from ICU Steps support group (for patients 6 or 12 months after an ICU stay). Those meeting inclusion criteria were provided with an envelope (if in hospital) or posted (if discharged) containing the study information and the SNAC questionnaire.

We conducted test/retest reliability by including a postcard asking participants to consider completing the questionnaire on a second occasion. On return of the postcard indicating consent, participants were posted a second copy of the questionnaire 2 weeks later.^35^ Both questionnaires were paired to assess test/retest reliability.

#### 2.2.4 Data analysis

Data analysis was conducted using IBM SPSS version 25 by the research team's statistical expert (ML). The number of questionnaires distributed and returned was recorded. Completed questionnaires were checked for missing items, free text, or errors. Data were summarized descriptively using numbers, percentages, means and standard deviations and explored for potential trends or correlations with clinical and demographic variables (gender, age, living arrangements, and employment status) to inform analysis of a subsequent longitudinal survey. We explored correlations with Pearson's coefficient and differences between questionnaire scores and patient demographics using one‐way ANOVA with post hoc comparisons using Tukey's HSD. Internal consistency (reliability) of questionnaire items was determined using Cronbach's alpha. Test–retest reliability was explored using intraclass correlation coefficients.

## 3 RESULTS

### 3.1 Stage 1. Questionnaire development and assessment of validity

Feedback from ICU service users and health care professionals indicated that the content of the questionnaire would help capture patients' post‐ICU support needs, and that most questions were easy to understand. There was consensus that most people would be able to complete this questionnaire following ICU discharge.

Based on feedback, some amendments were made to the questionnaire. In the section on emotional needs, two statements were combined and one statement was removed; in the section on appraisal needs, three statements were added and one removed; some wording was amended slightly to improve readability. The assessment of congruency found that the average percentage agreement between service users was 100% with 84% of questions. No items were rated unimportant. The research team reviewed the statements (5/31, 16%) where 100% congruency was not obtained and consensus was reached to retain these.

The Drivel Defence Index^33^ identified four statements with greater than 20 words out of the total 32 statements. We revised the questionnaire accordingly so that no statement had more than 20 words as recommended.

The final questionnaire (version 2.0 Supplementary Figures/Tables) comprised 32 statements addressing informational needs (n = 12); emotional needs (n = 9); instrumental needs (n = 6); appraisal needs (n = 4); and spiritual needs (n = 1). Respondents are asked to consider their current needs and rate agreement with each statement on a 5‐point Likert scale ranging from strongly disagree (scoring 1) to strongly agree (scoring 5).^25^ A total questionnaire score can be summated as well as a score for each category; higher scores reflect greater needs. We included space for open text after each support needs category to identify any additional needs not already included.^28^, ^36^ SNAC also included seven items to capture the sociodemographic and clinical characteristics of respondents.

### 3.2 Stage 2. Pilot survey to assess recruitment of ICU survivors at key stages of recovery, reliability of SNAC, and conduct exploratory analysis of data

#### 3.2.1 Recruitment and population characteristics

The recruitment period was December 2018 to April 2019. There were 375 support needs questionnaires distributed to eligible patients. We received n = 202/375 (54%) questionnaires: one was returned uncompleted by a family member as the patient was unwell, and one respondent did not meet inclusion criteria, resulting in a total of n = 200 questionnaires for analysis. Respondents were recruited from hospital registers (n = 197/200, 98%) and ICU steps (3/200, 2%).

The majority of respondents were male (n = 115, 57%); living with support from family members/carers (n = 154, 77%) and had not been offered a follow‐up appointment (n = 113/203 responses; 56%). Respondents were either still in hospital (n = 19, 9.5%); or discharged from hospital less than 6 weeks ago (n = 33, 16.5%); discharged from ICU between 7‐weeks and 6‐months (n = 71, 36%); 7 to 12 months (n = 51, 25.5%); or 1 to 2 years (n = 26, 13%). Respondent characteristics are summarized in Table 1. There were less than 5% missing responses from the potential total responses for the SNAC questionnaire. Given this low rate, the chances that these data had a major influence on our findings is minimal,^37^ indicating relevance and perceived importance of the items included. One statement pertaining to need for “information about returning to work” was missed by a 27/200, 14% of respondents. All data were analysed on a per protocol basis and no missing data were accounted for through statistical modelling.

**TABLE 1:** Characteristics of the study population

Characteristic	Number (%) participants
Gender	
Male	115 (57.5)
Female	85 (42.5)
Age years mean (SD)	57.53 (14.3)
Living arrangements	
Live alone	46 (23.0)
Live with family/friends/supported	154 (77.0)
Time since discharge	
Still in hospital	19 (9.5)
Less than 6 wk	33 (16.5)
7 wk to 6 mo	71 (35.5)
7 to 12 mo	51 (25.5)
1 to 2 y	26 (13.0)
Pre‐ICU employment status^a^	
Employed/voluntary/paid	79 (39.5)
Retired/unemployed/unable to work because of health	118 (59.0)
Ethnicity^b^	
White	190 (95.0)
Black / African / Caribbean / Black British	3 (1.5)
Asian / Asian British	1 (0.5)
Other ethnic group, please state	2 (1.0)
Prefer not to say	2 (1.0)
Follow‐up appointment offered	
Offered and attended	61 (29.0)
Offered but not attended	4 (2.0)
Not offered	113 (53.0)
Would have liked one	35 (16.0)

^a^ 3 did not respond.

^b^ 2 did not respond.

#### 3.2.2 Reliability and internal consistency of the SNAC questionnaire

Cronbach's alpha showed excellent reliability/internal consistency for the SNAC questionnaire overall (0.97) and for each category of need (information needs, 0.95; emotional needs, 0.95; instrumental needs, 0.93; and appraisal needs, 0.91). Test retest reliability questionnaires (n = 36) were completed by participants on two occasions approximately 2 weeks apart. Reliability of the total SNAC questionnaire (r = 0.8) was very good. Reliability of the five categories ranged from 0.3 to 0.9 (Supplementary Figures/Tables: Table 1).

#### 3.2.3 SNAC questionnaire exploratory results

**FIGURE 2:**
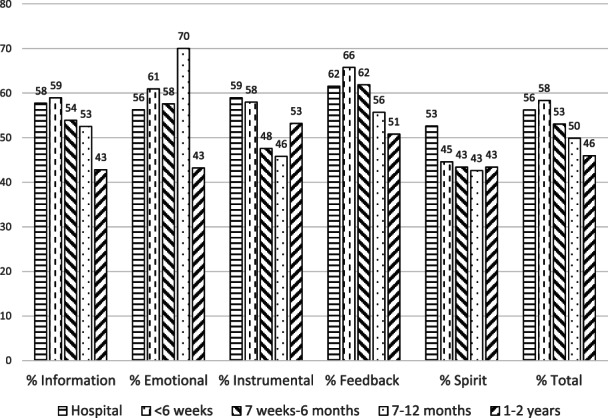
Proportion of patients with support needs (i.e. those who responded that they were undecided, or agreed, or strongly agreed they had specific support needs) in each of the categories at each recovery timepoint

Table 2 shows the mean (SD) overall scores for the total SNAC (range 32‐160) and each category; information (range 12‐60); emotional (range 9‐45); instrumental (range 6‐30); appraisal (range 4‐20); and spiritual (range 1‐5) within each of the five timepoints. Figure 2 shows the proportion of patients with support needs (i.e. those who responded that they were undecided, or agreed, or strongly agreed that they had specific support needs^25^) in each of the categories at each recovery timepoint. There was no significant SNAC score difference among any specific time point of recovery (F[4195] = 1.88, *P* = .12).

**TABLE 2:** Mean score for the SNAC questionnaire and for each needs category at each recovery timepoint after ICU discharge

Support need	SNAC	Informational needs	Emotional needs	Instrumental needs	Appraisal needs	Spiritual needs
Range	(32‐160)	(12‐60)	(9‐45)	(6‐30)	(4‐20)	(1‐5)
Mean (SD)	84 (32) N = 200	32 (14)	25 (11)	15 (8)	12 (5)	2 (1)
In hospital	90 (25) N = 19	35 (11)	25 (9)	18 (8)	12 (4)	3 (1)
<6 wk	93 (31) N = 33	35 (15)	27 (10)	17 (7)	13 (5)	2 (1)
7‐wk to 6‐mo	85 (32) N = 71	32 (14)	26 (10)	14 (8)	12 (5)	2 (1)
7 to 12 mo	80 (30) N = 51	32 (14)	23 (10)	14 (7)	11 (5)	2 (1)
1 to 2 y	74 (36) N = 26	27 (16)	20 (12)	16 (9)	10 (6)	2 (1)

#### 3.2.4 Gender and support needs

Between males and females, there were no significant differences in emotional, informational, appraisal, or spiritual needs. However, females had statistically significant higher (F[1198] = 4.66, *P* = .03) instrumental needs than males (mean (SD) males, 14.24 (7.54); females, 16.55 (7.39); mean difference (MD) 2.3).

Among each recovery timepoint for females, we found no significant differences in the support needs category scores (F[4,80] = 0.13, *P* = .97).

Among each recovery timepoint for males, information needs differed significantly (F[4110] = 2.85, *P* = .03) and this was higher for males at <6 weeks than 1 to 2 years (mean 37.6 versus 21.1, MD 16.5, 95% CI 2.51‐30.51, *P* = .01). There were also significant differences in emotional needs (F[4110] = 4.004, *P* = .01), higher at <6 weeks than 1 to 2 years (mean 30.3 versus 16.1, MD 14.17; 95% CI 4.29‐24.05; *P* = < .01); instrumental needs (F(4,110) = 3.35, *P* = .01, higher at <6 weeks than 7 weeks to 6 months, (mean 19.4 versus 12.4, MD 7.021, *P* = .01) and < 6 weeks and 7 to 12 months (19.4 versus 13.4, MD 6.0, *P* = .04). There were no significant differences in appraisal (F(4,110) = .458, *P* = .22) or spiritual needs (F[4103] = 0.58, *P* = .68).

#### 3.2.5 Age and support needs

Age and total SNAC scores were significantly correlated (r = −0.18, *P* = .01). Younger people had significantly more need for information (r = −0.14, *P* = .05 and emotional needs [r = −0.27, *P* < .00]. There were no significant correlations between age and instrumental needs r = −0.05, *P* = .53), appraisal needs (r = −0.11, *P* = .13) or spiritual needs (r = −0.047, *P* = .53).

#### 3.2.6 Living arrangements, employment status, and support needs

Among any recovery timepoint, there were no significant differences in support needs regardless of whether participants lived alone or with others; or whether participants were in employment/voluntary work before ICU and those who were retired/not working (see Supplementary Figures/Tables).

## 4 DISCUSSION

A key priority for the intensive care community is to identify how and when to support ICU survivors at key transitions in care, and throughout the recovery process. We used an iterative and robust process to develop a patient‐centred questionnaire to identify patient needs after admission to ICU. Postal distribution showed a moderate response rate. We have demonstrated that the questionnaire has good face and content validity, high internal consistency, and test–retest reliability. Because of sample size limitations, we were unable to assess construct validity, but will do so in subsequent work. Future work will also assess the questionnaire's ability to capture patients' evolving needs at repeated time points during recovery.

To date most services, when evaluated, generally use health‐related quality of life measures or changes in physical or psychological morbidity rather than patient‐identified needs.^14^, ^38^ To our knowledge, this is the first theoretically informed needs assessment questionnaire for ICU patients that can be used at different time points across the recovery trajectory.^4^, ^15^, ^22^, ^23^, ^24^, ^30^ It is patient‐focused and self‐completed, enabling patients to identify their social support needs across informational, emotional, instrumental, appraisal, and spiritual domains.

In the pilot survey, we received participant responses at each recovery time point. The lower number of participants recruited while still in hospital highlights a particular challenge as patients become orientated to the ward setting after ICU, and priorities focus on a speedy discharge.^39^ Specific recruitment strategies such as assistance for patients completing the SNAC questionnaire on the ward may need to be considered; if this is not possible, then completion as soon as possible after hospital discharge may still reflect early support needs. Similarly, the low number of participants recruited at 1 to 2 years could be reflective of undiscovered challenges at this stage of recovery, or conversely less support needs; or that specific strategies are needed to improve recruitment rates. Overall, the use of postal distribution with one postal reminder was efficient in this pilot study, but other additional strategies to enhance returns and optimize retention in a future longitudinal study will be necessary. A systematic review of flexible methods for data collection such as electronic completion, telephone completion, and/or home visits have been associated with improved retention rates in longitudinal studies.^40^

Results from the exploratory analysis show that the questionnaire captures support needs in the informational, emotional, instrumental, appraisal, and spiritual needs categories. The results also provide a signal indicating different needs may be based on patient characteristics and different time points (e.g. older people had less need for information and less emotional needs than younger people; males had greater information needs at <6 weeks than 1‐2 years). Future research with a more diverse population could explore this further as well as linkages between different clinical and socioeconomic factors which have been shown to influence recovery post ICU.^41^, ^42^, ^43^, ^44^, ^45^, ^46^ Additionally, it would be interesting to examine support needs and self‐efficacy, as greater self‐efficacy has been associated with recovery after ICU.^40^ Other new questionnaires are evolving which could assess long‐term health‐related quality of life after ICU and measure recovery.^38^, ^47^ Importantly, needs‐driven care should be based on patients' perceptions of need. This could provide important motivation and patient empowerment. Research in other health care populations and settings supports the use of needs assessment tools where patients can specifically identify support needs which may not otherwise be noted. These can enable health care professionals to recognize and determine appropriate care for a patient.^48^

### 4.1 Strengths and limitations

This study has a number of strengths. First, the use of an existing theoretical framework to identify support needs and key recovery stages after ICU.^4^, ^15^, ^22^, ^23^, ^24^, ^30^ Second, a robust and iterative process that included views from patients with experience of a stay in ICU throughout the development process.^25^, ^26^, ^27^, ^28^, ^29^ Third, the questionnaire facilitated patients to identify their own support needs^48^

The study also had a number of limitations. First, the response rate was 54%. It is, therefore, not possible to ascertain if nonresponders had higher, or no, support needs; it was too burdensome to complete; or they did not want to participate in research. Unlike general population surveys where response rates less than 70% are considered at risk of bias,^49^ low response rates are predominantly a methodological limitation of undertaking postal surveys in this critical care population.^50^

Second, because of financial and time‐resource limitations, we were unable to follow patients over time to assess whether and how their needs evolved. The transition from intensive care through to recovery and survivorship follows an individualized timeline and further insight into patient's evolving needs could influence care pathways.^51^ Third, 96% of respondents identified themselves as “White” limiting the generalizability of the results to Black and Asian minority ethnic groups. Future work should address recruitment strategies by using the INCLUDE Ethnicity Framework. This framework helps researchers consider which ethnic groups should be included to ensure results are widely applicable, and the challenges in making this possible.^52^ Fourth, the timeframes may be considered wide, e.g. the stage between 7 weeks and 6 months, as patients' needs may change during this time. However, the timelines for assessment of the 5 stages of recovery were broadly aligned to the TIR framework which we had used for the study from the outset.

## 5 CONCLUSION

The SNAC questionnaire appears to be a comprehensive, valid, and reliable questionnaire that could help to identify ICU survivors' support needs. Although there are some limitations of the pilot survey, the SNAC questionnaire has the potential to discover which support services need to be available for ICU survivors. Subsequent research will enable a more robust examination of its psychometric properties e.g. factor analysis, and establish its utility in identifying whether patients' support needs evolve over time. Future work may also need to explore the extent to which patients feel able to engage with support services even when these are available.

## ETHICS STATEMENT

Ethical approval was granted from the Office for Research Ethics Northern Ireland (Reference 17/NI/0236) and research governance for individual collaborating sites was obtained.

## PATIENT CONSENT STATEMENT

The return of the completed questionnaire will be accepted as consent. Additionally, we conducted test/retest reliability by including a postcard asking participants to consider completing the questionnaire on a second occasion. On return of the postcard indicating consent, participants were posted a second copy of the questionnaire 2 weeks later.

## Supporting information


**Appendix S1:** Supporting Information

## Data Availability

The data that support the findings of this study are available from the corresponding author upon reasonable request.
