# Supplementary material for: Development of the support needs after ICU (SNAC) questionnaire
Source: Nurs Crit Care. 2021 Aug 13;27(3):410–8. doi: 10.1111/nicc.12695 (PMC9290803; doi:10.1111/nicc.12695)
Supplement: Supplementary file 1 — Appendix S1: Supporting Information [file NICC-27-410-s001.doc]

**Supplementary Figures/Tables**

Section 1. Service User Panel and Expert panel Feedback Sheets for Needs Assessment Questionnaire

Section 2. Service User Panel Feedback Sheet for Needs Assessment Questionnaire Congruency and Readability

Section 3. SNAC questionnaire

Section 4. Pilot study - Additional results

Section 1.

Service User Panel and Expert panel Feedback Sheets for Needs Assessment Questionnaire

**Service User Panel**

Feedback Sheet for Needs Assessment Questionnaire

**Please indicate how strongly you agree or disagree with the following statements by placing a tick in the box that corresponds to your answer.**

**1 = strongly disagree 5 = strongly agree**

| **Q1.** | I think this questionnaire would help us determine what people need when they come out of ICU. | 1 | 2 | 3 | 4 | 5 |
| --- | --- | --- | --- | --- | --- | --- |

Comments: ___________________________________________________________

**1 = strongly disagree 5 = strongly agree**

| **Q2.** | I think the questions were easy to understand. | 1 | 2 | 3 | 4 | 5 |
| --- | --- | --- | --- | --- | --- | --- |

Comments: ___________________________________________________________

**1 = strongly disagree 5 = strongly agree**

| **Q3.** | There were some questions that I needed help with or further clarification. | 1 | 2 | 3 | 4 | 5 |
| --- | --- | --- | --- | --- | --- | --- |

Comments: ___________________________________________________________

**1 = strongly disagree 5 = strongly agree**

| **Q4.** | I felt confident that I could fill out this questionnaire myself. | 1 | 2 | 3 | 4 | 5 |
| --- | --- | --- | --- | --- | --- | --- |

Comments: ___________________________________________________________

**1 = strongly disagree 5 = strongly agree**

| **Q5.** | I would imagine that most people would be able to fill out this questionnaire when out of ICU. | 1 | 2 | 3 | 4 | 5 |
| --- | --- | --- | --- | --- | --- | --- |

Comments: ___________________________________________________________

**1 = strongly disagree 5 = strongly agree**

| **Q6.** | I was generally satisfied with the layout and format of the questionnaire. | 1 | 2 | 3 | 4 | 5 |
| --- | --- | --- | --- | --- | --- | --- |

Comments: ___________________________________________________________

| **Q7.** | Is there anything you would add to or remove from the questionnaire? | YES | NO |
| --- | --- | --- | --- |

Comments: ___________________________________________________________

| **Q8**. | Do you think the questionnaire was | | |  |
| --- | --- | --- | --- | --- |
|  | 🞎 Too long | 🞎 Too short | 🞎 About right |  |

**Thank you for taking the time to complete this questionnaire. If you would like to make any further comments please do so in the box below.**

**Expert Advisory Group**: Feedback Sheet for Needs Assessment Questionnaire

**1 = strongly disagree 5 = strongly agree**

| **Q1.** | In general, I think this questionnaire would help clinicians determine what people need after a stay in ICU | 1 | 2 | 3 | 4 | 5 |
| --- | --- | --- | --- | --- | --- | --- |

Comments:

| **Q2.** | Do you feel strongly that there are questions or themes that still need added to the questionnaire? | No | Yes |
| --- | --- | --- | --- |

If yes, please indicate the questions/themes here:

**1 = strongly disagree 5 = strongly agree**

| **Q3.** | I think that there are some questions that may need further clarification | 1 | 2 | 3 | 4 | 5 |
| --- | --- | --- | --- | --- | --- | --- |

Comments/questions that need further clarification:

| **Q4.** | Are there any questions or themes you feel we could remove from the questionnaire if we need to reduce the content? | YES | NO |
| --- | --- | --- | --- |

Comments/questions that we could remove if needed:

**1 = strongly disagree         5 = strongly agree**

| **Q5.** | I would imagine that most people would be able to fill out this questionnaire when out of ICU. | 1 | 2 | 3 | 4 | 5 |
| --- | --- | --- | --- | --- | --- | --- |

Comments:

**1 = strongly disagree         5 = strongly agree**

| **Q6.** | I was generally satisfied with the layout and format of the questionnaire. | 1 | 2 | 3 | 4 | 5 |
| --- | --- | --- | --- | --- | --- | --- |

Comments:

| **Q7**. | Do you think the questionnaire is | | |  |
| --- | --- | --- | --- | --- |
|  | 🞎 Too long | 🞎 Too short | 🞎 About right |  |

Any other comments/notes that we could consider

Section 2

Service User Panel Feedback Sheet for Needs Assessment Questionnaire Congruency and Readability

**Service User Panel**

Feedback Sheet for Needs Assessment Questionnaire including congruency

| **After looking at the questionnaire, please rate how important you think each item is: 1 = very important, this item should definitely be included in the questionnaire; 2 = somewhat important, it would not matter if this item was in or out of the questionnaire; 3 = not important, I think this item should be removed from the questionnaire** | | | | | |
| --- | --- | --- | --- | --- | --- |
| ***At this point in time, I need information about…*** | ***Rating*** | ***At this point in time, I need help in relation to my emotions about…*** | ***Rating*** | ***At this point in time, I need assistance…*** | ***Rating*** |
| Why I came into ICU |  | How I feel at this time |  | From others to carry out everyday care for me |  |
| What happened when I was in ICU |  | Why I came into ICU and what happened when I was there |  | To look after myself |  |
| What to expect in the next steps of my recovery |  | Moving to different settings |  | For either getting around the house or outside |  |
| What I can and cannot do |  | The attention I received from healthcare staff |  | To get access to other therapies |  |
| Coping with my physical needs |  | The attention I need from friends/family |  | To do household activities |  |
| Physical aids I might need |  | The next steps in my recovery |  | More training or explanation with using the equipment aids supplied |  |
| Medication |  | The impact of my illness on my family and friends |  | ***At this point in time, I need feedback on my overall progress…*** | ***Rating*** |
| My physical appearance |  | The impact of my illness on my finances |  | From healthcare professionals |  |
| Strange feelings |  | The impact of my illness on returning to work |  | From my family or friends |  |
| Financial support |  | The impact of my illness on returning to usual activities |  | I need a face-to-face follow-up appointment with the ICU team |  |
| Returning to work |  |  |  | ***At this point in time…*** | ***Rating*** |
| Returning to my usual activities |  |  |  | I need spiritual support |  |

**Readability, understanding and user-friendliness**

**Please circle yes or no to indicate your answer to the following questions:**

1. Were you able to easily read all the words used? **Yes / No**

If no, which words did you find hard to read? _____________________

_________________________________________________________

_________________________________________________________

1. Did you find any of the items hard to understand? **Yes / No**

If yes, which items did you find hard to understand? ________________

_________________________________________________________

_________________________________________________________

1. Did you find the questionnaire easy to complete/follow? **Yes / No**

If no, please explain why _____________________________________

_________________________________________________________

_________________________________________________________

1. Please use the space below to add any other comments about the questionnaire you would like to share with the team?

_________________________________________________________

_________________________________________________________

_________________________________________________________

Section 3

**S**upport **N**eeds **A**fter Intensive **C**are **(SNAC)**

***Please give us a little information about yourself***

1. **Are you**

🞎 Male 🞎 Female

1. **What age are you? ______________ (Years)**
2. **Do you**

🞎 live alone 🞎 live with family/friends/supported living/other

1. **Were you in work before your intensive care unit (ICU) stay?**

🞎 Yes (employed/self-employed/full time/part time/voluntary/paid)

🞎 No (retired/unemployed/unable to work due to health)

1. **How long is it since you were discharged from the hospital?**

🞎 I am still in hospital

🞎 Less than 6 weeks ago

🞎 Between 7 weeks and 6 months ago

🞎 Between 7 and 12 months ago

🞎 Between 1 year and 2 years ago

1. **How would you describe your ethnicity?**

🞎 White 🞎 White and Black Caribbean

🞎 Black / African / Caribbean / Black British 🞎 White and Black African

🞎 Asian / Asian British 🞎 White and Asian

🞎 Other ethnic group, please state ____________________________

🞎 Prefer not to provide

1. **After you went home from hospital, were you offered a follow-up appointment to see your ICU team? (Please tick appropriate)**

🞎 Offered and attended

🞎 Offered but did not attend

🞎 Not offered

🞎 Would have liked one

| **S**upport **N**eeds **A**fter Intensive **C**are **(SNAC)**  **This questionnaire aims to find out about your needs for support and services after a stay in the intensive care unit (ICU). Please rate how much you agree with each statement. You may have other needs not included in the questionnaire. If so, please add them in the space at the end of each section.** | | | | | |
| --- | --- | --- | --- | --- | --- |
| ***At this point in time, I need…*** | Strongly Disagree | Disagree | Undecided | Agree | Strongly Agree |
| Information about why I came into ICU |  |  |  |  |  |
| Information about what happened when I was in ICU |  |  |  |  |  |
| Information about what to expect in the next steps of my recovery |  |  |  |  |  |
| Information about coping with my physical needs  (e.g. wounds, fatigue, loss of appetite) |  |  |  |  |  |
| Information about changes in the way I look |  |  |  |  |  |
| Information about equipment I might need |  |  |  |  |  |
| Information about medication  (e.g. what it is for, when to take it, review of medications, side effects) |  |  |  |  |  |
| Information about strange feelings  (e.g. dreams, memories of time in ICU) |  |  |  |  |  |
| Information about what I can and cannot do  (e.g. getting up and about, using bathroom, sleeping, eating) |  |  |  |  |  |
| Information about getting back to my usual activities  (e.g. swimming, sex, walking, driving) |  |  |  |  |  |
| Information about returning to work |  |  |  |  |  |
| Information about financial support  (e.g. disability allowances, social benefits) |  |  |  |  |  |
| **Tell us about other information you need** | | | | | |

| ***At this point in time, I need help…*** | Strongly Disagree | Disagree | Undecided | Agree | Strongly Agree |
| --- | --- | --- | --- | --- | --- |
| Coping with how I feel at this time  (e.g. sadness, low mood, anxiety, fear) |  |  |  |  |  |
| Coping with my feelings about being in the ICU |  |  |  |  |  |
| Coping with my feelings about the next steps in my recovery |  |  |  |  |  |
| Coping with my feelings about moving to different settings  (e.g. from ICU to the ward, from ward to home) |  |  |  |  |  |
| Coping with my feelings about the care and attention I received from healthcare staff |  |  |  |  |  |
| Coping with my feelings about the support I need from friends and family |  |  |  |  |  |
| Coping with my feelings about the impact of my illness on my family and friends  (e.g. emotional, physical, caring roles) |  |  |  |  |  |
| Coping with my worries about money |  |  |  |  |  |
| Getting back to my usual self |  |  |  |  |  |
| **Tell us about other feelings you need help with** | | | | | |

| ***At this point in time, I need …*** | Strongly Disagree | Disagree | Undecided | Agree | Strongly Agree |
| --- | --- | --- | --- | --- | --- |
| Help to look after myself  (e.g. bathing, washing hair, going to the toilet, mouthcare, feeding myself) |  |  |  |  |  |
| Help from others to carry out everyday care for me |  |  |  |  |  |
| Help to do household activities |  |  |  |  |  |
| Help for either getting around inside **or** outside the house |  |  |  |  |  |
| Help to get other therapies |  |  |  |  |  |
| More training or explanation with using any equipment supplied |  |  |  |  |  |
| **Tell us about other practical help you need** | | | | | |

| ***At this point in time, I need…*** | Strongly Disagree | Disagree | Undecided | Agree | Strongly Agree |
| --- | --- | --- | --- | --- | --- |
| Feedback on how far I’ve come and where I should be now |  |  |  |  |  |
| Feedback on my progress from healthcare professionals |  |  |  |  |  |
| Feedback on my progress from my family or friends |  |  |  |  |  |
| My family to be aware of my progress |  |  |  |  |  |
| **Tell us about other feedback you need** | | | | | |

| ***At this point in time…*** | Strongly Disagree | Disagree | Undecided | Agree | Strongly Agree |
| --- | --- | --- | --- | --- | --- |
| I need spiritual support |  |  |  |  |  |

Section 4: Additional Results

Supplementary Table 1 SNAC questionnaire Test-retest reliability

|  | Correlation coefficient |
| --- | --- |
| **SNAC total** | **0.79** |
| Informational needs | 0.81 |
| Emotional needs | 0.54 |
| Instrumental needs | 0.87 |
| Appraisal needs | 0.57 |
| Spiritual needs | 0.27 |

### Additional results for “Do support needs differ depending on respondent characteristics”

*Living arrangements and support needs*

When the dataset was split by living status there were no statistically significant differences found for those who lived alone [informational F(4,41) = .904, p = .471; emotional F(4,41) = .722, p = .116; instrumental F(4,41) = .679, p = .611; appraisal F(4,41) = .846, p = .504; spiritual F(4,37) = .223, p = .924; total needs F(4,41) = .602, p = .663] and those who lived with others [informational F(4,149) = 1.681, p = .157; emotional F(4,149) = 1.884, p = .116; instrumental F(4,149) = 1.594, p = .179; appraisal F(4,149) = 1.224, p = .303; spiritual F(4,140) = .874, p = .481; total needs F(4,149) = 1.641, p = .167] at any time point.

#### Home/employment and support needs

Among any TIR time cohort, there were no significant differences in support needs regardless of whether participants lived alone or with others; or whether participants were in employment/voluntary work before ICU and those who were retired/not working.

The dataset was then split by occupation to show no statistically significant differences for those in employment/voluntary work before ICU [informational F(4,75) = 1.283, p = .284; emotional F(4,75) = .774, p = .565; instrumental F(4,75) = .932, p = .450; appraisal F(4,75) = .257, p = .904; spiritual F(4,68) = .232, p = .919; total needs F(4,74) = .933, p = .449] and those who were retired/not working [informational F(4,112) = .662, p = .620; emotional F(4,112) = 1.579, p = .185; instrumental F(4,112) = 1.160, p = .332; appraisal F(4,112) = 1.324, p = .265; spiritual F(4,106) = .739, p = .567; total needs F(4,113) = .855, p = .494] at any time point.
